# Two types of Wechsler Adult Intelligence Scale (WAIS) index discrepancies are associated with insufficient weight reduction after sleeve gastrectomy in adult patients with obesity: a retrospective study

**DOI:** 10.1186/s40001-025-03758-y

**Published:** 2026-01-06

**Authors:** Masahiro Ohira, Sayaka Tsuji, Yasuhiro Watanabe, Osamu Horikawa, Naoyuki Kawagoe, Taiki Nabekura, Kozue Hashi, Karin Hayashi, Daiji Nagayama, Ichiro Tatsuno, Takashi Oshiro, Atsuhito Saiki

**Affiliations:** 1 Division of Diabetes, Metabolism and Endocrinology, Department of Internal Medicine, Toho University Ohashi Medical Center, 2-22-36 Ohashi, Meguro-Ku, Tokyo, 153-8515 Japan; 2 Center for Diabetes, Endocrine and Metabolism, Toho University Sakura Medical Center, Chiba, Japan; 3 Department of Surgery, Toho University Sakura Medical Center, Chiba, Japan; 4 Department of Neuropsychiatry, Toho University Sakura Medical Center, Chiba, Japan; 5 Department of Internal Medicine, Nagayama Clinic, Tochigi, Japan; 6 Chiba Prefectural University of Health Sciences, Chiba, Japan; 7 Department of Surgery, The Jikei University School of Medicine, Tokyo, Japan

**Keywords:** Obesity, Laparoscopic sleeve gastrectomy, Intelligence quotient, Discrepancy, Excess weight loss

## Abstract

**Introduction:**

Obesity is a major global health concern. Laparoscopic sleeve gastrectomy (LSG) has demonstrated excellent therapeutic effects. Further, intelligence quotient (IQ) is an important factor in obesity; however, full-scale IQ is not associated with weight reduction after LSG. IQ and IQ index score discrepancies are also important when considering IQ tests. However, the relationship between IQ or IQ index score discrepancies and weight reduction after bariatric surgery remains unexplored. This study aimed to elucidate the relationship between IQ index score discrepancies and weight reduction following LSG.

**Methods:**

In total, 204 patients with obesity who underwent LSG and were followed up for 12 months were retrospectively reviewed. The relationship between IQ, the Wechsler Adult Intelligence Scale (WAIS) index score discrepancy, and various clinical parameters, particularly weight reduction, after LSG was analyzed. A logistic regression analysis was performed.

**Results:**

The percentage of excess weight loss (%EWL) was 64.2 (49.6–84.5%) 12 months post-LSG. IQ scores were not associated with preoperative body mass index. However, patients with WAIS index score discrepancies between the perceptual reasoning index (PRI), processing speed index (PSI), and working memory index (WMI) had low %EWL. Logistic regression analysis revealed that the existence of WAIS index score discrepancies between the PRI/WMI and PSI was associated with low %EWL post-LSG. Further, patients with WAIS index score discrepancies in the PRI > PSI group had significantly lower %EWL than those in the PRI < PSI group.

**Conclusion:**

These results show that the existence of WAIS index score discrepancies between the PRI/WMI and PSI is associated with insufficient weight reduction after LSG in patients with obesity.

**Supplementary Information:**

The online version contains supplementary material available at 10.1186/s40001-025-03758-y.

## Introduction

Obesity is now considered a major global health concern, associated with various risk factors for atherosclerotic diseases, including type 2 diabetes, lipid disorders, and hypertension. Bariatric surgery is currently the most effective weight loss therapy, with laparoscopic sleeve gastrectomy (LSG) being the most commonly performed procedure. LSG leads to an average 69.7% excess weight loss (EWL) 1 year postoperatively [1]. Its effects on weight reduction and obesity-related diseases, such as type 2 diabetes and lipid disorders, are superior to those associated with lifestyle modifications and non-surgical treatments [2–4]. Furthermore, LSG is associated with low complication rates, with perioperative and serious complications occurring in 5.4% and 1.2% of cases, respectively [5]. Thus, LSG is considered a safe treatment owing to its excellent therapeutic effects on obesity and obesity-related diseases. Therefore, the IFSO/ASMBS guidelines recommend metabolic bariatric surgery for patients with a BMI of 30–34.9 kg/m^2^ and one obesity-associated medical problem [6].

Intelligence quotient (IQ) is an important factor in obesity. Low IQ is associated with the prevalence of obesity in adolescence [7], and obesity is, in turn, associated with increased prevalence of low cognitive function, as evaluated by IQ tests in young men [8]. In contrast to these findings in population-based studies, previous research in bariatric surgery candidates has shown that full-scale IQ and IQ index scores are not associated with body mass index (BMI) or body weight (BW) in adolescents with obesity seeking laparoscopic adjustable gastric banding [9], and that full-scale IQ is also not associated with weight reduction following LSG [10]. Despite this, IQ or IQ index score discrepancy is important when considering IQ tests. The IQ discrepancy between verbal and performance IQ influences fine motor skills and motor competence [11, 12]. Further, a discrepancy in the Wechsler Adult Intelligence Scale (WAIS) index score has been reported between patients with attention deficit hyperactivity disorder and controls [13]. However, the relationship between IQ or IQ index score discrepancies and obesity remains unexplored. We hypothesized that IQ index score discrepancies would be associated with insufficient postoperative weight loss. Therefore, this study aimed to investigate the relationship between IQ, including IQ index score discrepancy, and weight reduction following LSG in adults with obesity.

## Methods

### Study design and participants

This single-center retrospective study reviewed patients who underwent LSG between July 2010 and November 2023 at Toho University Sakura Medical Center (Sakura City, Chiba, Japan). Inclusion criteria were: a diagnosis of primary obesity (BMI ≥ 32 kg/m^2^ at the initial visit) and completion of LSG, with 12 months postoperative follow-up. Exclusion criteria were: absence of preoperative IQ assessment, non-native Japanese speakers (as the IQ test was conducted in Japanese), and withdrawal from follow-up within 12 months post-LSG.

In Japan, obesity is defined as a BMI ≥ 25.0 kg/m^2^ [14]. According to the Japanese Society for the Treatment of Obesity, eligibility for bariatric surgery includes either a BMI ≥ 32 kg/m^2^ with comorbidities (type 2 diabetes mellitus, hypertension, or hyperlipidemia) or a BMI ≥ 35 kg/m^2^ regardless of comorbidities. In this study, 189 patients had a BMI ≥ 35 kg/m^2^. Among them, 32 patients had no comorbidities. In total, 255 LSG cases were reported during the study period. However, 51 patients were excluded: 18 (7.1%) withdrew within 12 months postoperatively, 25 (9.8%) did not undergo preoperative IQ tests, and 8 (3.1%) were non-native Japanese speakers. Ultimately, 204 adult patients with obesity (80.0%) who underwent LSG were included in this study. The study size was determined by including all consecutive patients who met the eligibility criteria during the study period; no formal sample size calculations were performed. There were no significant differences in sex, age, preoperative BW, preoperative BMI, prevalence of type 2 diabetes, or psychiatric comorbidity between included and excluded patients (Supplementary Table 1).

We attempted to minimize selection bias by including all consecutive eligible patients during the study period. Measurement bias was reduced by performing all anthropometric and biochemical assessments using standardized procedures at a single center. Potential confounding factors were addressed by adjusting for relevant covariates in multivariate logistic regression models.

The following parameters were compared pre- and 12 months post-LSG: BW and BMI. Patients with glycosylated hemoglobin (HbA1c) and fasting blood glucose (FBG) levels ≥ 6.5% and 126 mg/dL, respectively, or already receiving anti-hypoglycemic agents, were diagnosed with type 2 diabetes. Psychiatrists diagnosed patients with mental disorders during IQ tests. Further, the percentage of excess weight loss (%EWL) was estimated at 12 months post-LSG. The %EWL was estimated as follows: (BW before surgery – BW 12 months after LSG)/(BW before surgery – BW corresponding to a BMI of 25 kg/m^2^ for each participant). Weight loss outcomes were evaluated 12 months after surgery, as this time point is commonly the maximal and stable weight reduction following bariatric surgery [15, 16], before the potential influence of long-term weight regain [17]. There were no missing data for the variables used in the primary analyses; therefore, complete-case analysis was conducted.

In 1982, Reinhold proposed that a %EWL value ≥ 50% indicated a “good” bariatric weight loss result [18]. Moreover, some clinical studies have defined insufficient weight loss after bariatric surgery as a %EWL < 50% [19–22]. Therefore, we categorized patients into two groups: patients with %EWL < 50% (n = 53, 26.0%) and those with %EWL ≥ 50% (n = 151, 74.0%), to compare patients with insufficient and sufficient weight reduction.

### IQ examination

IQ tests were performed using the WAIS, a standardized battery designed to evaluate intellectual ability, and were administered within 3 months before surgery. During this study, the WAIS was updated from version III to IV in our hospital in March 2019; however, the WAIS-IV has a very similar subtest and index structure as its predecessor, WAIS-III [23, 24]. Therefore, patients were analyzed using both the WAIS-III and WAIS-IV. However, verbal and performance IQ were not analyzed, as they were components of the WAIS-III and are no longer included in the WAIS-IV. In total, 86 (42.2%) and 118 (57.8%) patients were tested using the WAIS-III and WAIS-IV, respectively. Both were administered and scored according to the standardized procedures outlined in the manual [23, 25]. The primary WAIS-IV subtests yield four index scores (verbal comprehension index [VCI], perceptual reasoning index [PRI], working memory index [WMI], and processing speed index [PSI]), and an overall full-scale IQ score [25]. The WAIS-III expresses these indices as the VCI, perceptual organization index, WMI, and PSI [23]. To avoid confusion arising from different expressions in each WAIS index, we expressed the WAIS indices as VCI, PRI, WMI, and PSI, in accordance with a previous study [26]. The definitions of VCI, PRI, WMI, and PSI are described in Supplementary File 1 [27]. The WAIS divides IQ scores into subtests: extremely low (< 70), borderline (70–79), low average (80–90), average (90–109), high average (110–119), superior (120–129), and very superior (≥ 130) [23, 25]. Discrepancies in WAIS index scores were defined according to the standardized statistical criteria provided in the WAIS-III and WAIS-IV manuals [23, 25]. For each participant, we calculated the absolute difference between two index scores. The WAIS manuals provide age-specific critical values representing the minimum difference required for two index scores to be considered statistically significant at the 5% level [23, 25]. When the observed index difference exceeded the corresponding critical value, the pair of index scores was classified as demonstrating a "discrepancy".

### Statistical analysis

The normality of the data distribution was assessed using the Shapiro–Wilk test. As many variables were non-normally distributed, continuous data were expressed as medians and interquartile ranges (IQR). Data were analyzed using the Wilcoxon signed-rank test (paired samples) or the Wilcoxon rank-sum test (independent samples). Fisher’s exact test was used to identify significant differences between proportions and categorical variables. Patients were categorized into two groups based on the median preoperative BMI: BMI < 43.6 kg/m^2^ and ≥ 43.6 kg/m^2^, or based on %EWL post-LSG: %EWL < 50% and ≥ 50% according to the definition of insufficient %EWL [18–22]. Multivariate logistic regression analysis was used to identify the parameters contributing to a low %EWL (< 50%), with values expressed as odds ratios (ORs) and 95% confidence intervals (CIs). Significant differences were observed between the %EWL < 50% and %EWL ≥ 50% groups in sex, BW, BMI, VCI, and two types of WAIS index discrepancies (PRI–PSI and WMI–PSI) (Table 2). BW and BMI were analyzed as continuous variables. VCI scores were divided into two groups based on a clinically meaningful cut-off of 90. After adjusting for dichotomous confounders and confirming the absence of multicollinearity, sex, BW, BMI, VCI, and WAIS index score discrepancies (PRI–PSI and WMI–PSI) were included in the model. The results are presented as odds ratios (ORs) with 95% confidence intervals (CIs) and *P*-values (Table 3). To address potential measurement differences between WAIS-III and WAIS-IV, we additionally conducted multivariate logistic regression analyses separately for patients assessed with WAIS-III and those assessed with WAIS-IV. Statistical significance was set at* P* < 0.05. All statistical analyses were performed using the JMP Pro software (version 17.2.0; SAS Institute, Cary, NC, USA).

## Results

### Baseline characteristics and changes in various parameters 12 months post-LSG

Table 1 details the baseline characteristics and changes in patient parameters 12 months post-LSG. The median (IQR) age and BMI were 44.5 (37.0–50.0) years and 43.6 (38.7–50.4) kg/m^2^, respectively. In addition, 120 patients (58.8%) were diagnosed with type 2 diabetes mellitus. At 12 months post-LSG, the median %EWL was > 50%, 64.2 (49.6–84.5)%. BW and BMI significantly decreased post-LSG (Table 1). In total, 123 (60.3%) patients were diagnosed with mental disorders (Table 1). The median (IQR) full-scale IQ, VCI, PRI, and WMI scores were > 90, whereas the PSI score was < 90 (Table 1). The proportion of patients with discrepancies in each WAIS index score ranged from 40–50% (Table 1).

**Table 1:** Baseline characteristics and changes in various parameters at 12 months post-LSG

	Baseline	After 12 months	*P* value^a^
Sex (male/female)	95 (46.6%)/109 (53.4%)		
Age (years)	44.5 (37.0–50.0)		
Type 2 diabetes mellitus	120 (58.8%)		
BW (kg)	117.9 (101.2–138.7)	84.4 (72.0–101.1)	< 0.0001
BMI (kg/m^2^)	43.6 (38.7–50.4)	31.0 (27.6–36.9)	< 0.0001
%Excess weight loss (%)		64.2 (49.6–84.5)	
Patients with mental disorders	123 (60.3%)		
Depression	59 (28.9%)		
Anxiety disorders	23 (11.3%)		
Mental retardation/Developmental retardation	21 (10.3%)		
Sleeplessness	16 (7.8%)		
Bipolar disorder	14 (6.9%)		
Schizophrenia	9 (4.4%)		
Binge eating	9 (4.4%)		
Others	17 (8.3%)		
Full-scale IQ	92.0 (81.3–104.0)		
VCI score	96.0 (82.3–106.0)		
PRI score	93.0 (83.0–106.0)		
WMI score	92.5 (82.0–101.5)		
PSI score	89.0 (79.5–97.0)		
Patients with WAIS index score discrepancy			
VCI–PRI	82 (40.2%)		
VCI–WMI	94 (46.1%)		
VCI–PSI	100 (49.0%)		
PRI–WMI	84 (41.2%)		
PRI–PSI	92 (45.1%)		
WMI–PSI	83 (40.7%)		

Data are presented as medians, interquartile ranges (IQR), or percentages

^a^Wilcoxon signed-rank test

*LSG* laparoscopic sleeve gastrectomy, *IQ* intelligence quotient, *WAIS* Wechsler Adult Intelligence Scale, *VCI* verbal comprehension index, *PRI* perceptual reasoning index, *WMI* working memory index, *PSI* processing speed index

Other patients had obsessive–compulsive disorder (two patients), adjustment disorder (two patients), dissociative disorder, mood disorder, and alexithymia

### Comparison of characteristics and IQ examination between patients without and with mental disorders

Neither %EWL nor the proportion of patients with %EWL < 50% differed between those with and without mental disorders. Although patients with mental disorders had significantly lower full-scale IQ and PRI scores than those without mental disorders, the prevalence of mental or developmental retardation was significantly higher in patients with mental disorders. No significant differences were observed in VCI, WMI, PSI scores, or WAIS index score discrepancies between both groups (Supplementary Table 2).

### Comparison of characteristics, full-scale IQ, WAIS index scores, and WAIS index score discrepancies between preoperative BMI < 43.6 kg/m^2^ and ≥ 43.6 kg/m^2^ groups

Among patients with BMI < 43.6 kg/m^2^, %EWL post-LSG was significantly higher and the proportion of those with %EWL < 50% was significantly lower compared to patients with BMI ≥ 43.6 kg/m^2^ (Supplementary Table 3). No relationship was observed between preoperative BMI and full-scale IQ, WAIS index scores, or WAIS index score discrepancies (Supplementary Table 3).

### Comparison of characteristics between %EWL < 50% and ≥ 50%

In total, 53 (26.0%) and 151 (74.0%) patients were included in the %EWL < 50% and ≥ 50% groups, respectively. The distributions of sex, BW, BMI, VCI score, and the two types of WAIS index score discrepancies (PRI–PSI and WMI–PSI) differed between the two groups. The proportion of males, preoperative BW and BMI, and VCI score were significantly higher in the %EWL < 50% group than in the %EWL ≥ 50% group. However, %EWL was significantly lower in the %EWL < 50% group than in the %EWL ≥ 50% group (Table 2). No significant between-group differences were observed in the other parameters.

**Table 2:** Comparison of characteristics between %EWL < 50% and ≥ 50%

	%EWL < 50%	%EWL ≥ 50%	*P* value
No. of participants	53 (26.0%)	151 (74.0%)	
Sex (male/female)	34 (64.2%)/19 (35.8%)	61 (40.4%)/90 (59.6%)	0.0038^a^
Age (years)	45.0 (38.0–52.5)	44.0 (37.0–49.0)	0.1381^b^
Type 2 diabetes mellitus	33 (62.3%)	87 (57.6%)	0.6276^a^
Patients with mental disorders	35 (66.0%)	88 (58.3%)	0.3338^a^
Patients with depression	17 (32.1%)	42 (27.8%)	0.5988^a^
BW (kg)	135.4 (111.9–157.9)	114.2 (99.7–133.5)	0.0017^b^
BMI (kg/m^2^)	47.9 (41.9–55.2)	42.5 (38.0–47.9)	0.0006^b^
%EWL (%)	37.8 (27.6–45.5)	74.9 (61.3–91.7)	< 0.0001^b^
Full-scale IQ	98.0 (82.5–108.0)	91.0 (81.0–102.0)	0.0510^b^
VCI score	100.0 (90.0–110.5)	95.0 (82.0–102.0)	0.0136^b^
PRI score	99.0 (83.5–110.0)	93.0 (82.0–105.0)	0.1310^b^
WMI score	94.0 (82.0–107.0)	92.0 (82.0–100.0)	0.3106^b^
PSI score	87.0 (81.5–99.5)	89.0 (79.0–96.0)	0.5310^b^
Patients with WAIS index score discrepancy			
VCI–PRI	22 (41.5%)	60 (39.7%)	0.8712^a^
VCI–WMI	25 (47.2%)	69 (45.7%)	0.8739^a^
VCI–PSI	28 (52.8%)	72 (47.7%)	0.5280^a^
PRI–WMI	26 (49.1%)	58 (38.4%)	0.1963^a^
PRI–PSI	33 (62.3%)	59 (39.1%)	0.0040^a^
WMI–PSI	29 (54.7%)	54 (35.8%)	0.0224^a^

Data are presented as medians, interquartile ranges (IQR), or percentages

^a^Fisher’s exact test; ^b^Wilcoxon rank-sum test

*EWL* excess weight loss, *BW* body weight, *BMI* body mass index, *IQ* intelligence quotient, *WAIS* Wechsler Adult Intelligence Scale, *VCI* verbal comprehension index, *PRI* perceptual reasoning index, *WMI* working memory index, *PSI* processing speed index

### ORs [95% CIs] for low %EWL (%EWL ≥ 50%, 0; %EWL < 50%, 1) as analyzed by a multivariate logistic regression model

The contribution of each parameter to low %EWL post-LSG was examined using multivariate logistic regression models (Table 3). The ORs of %EWL < 50% were 2.09 (95% CIs 1.05–4.22, *P* = 0.0346) for the existence of WAIS index score discrepancy between PRI and PSI, and 1.97 (95% CIs 1.01–3.91, *P* = 0.0482) for the existence of WAIS index score discrepancy between WMI and PSI (Table 3). In the sensitivity analyses stratified by WAIS version, the odds ratios for both PRI–PSI and WMI–PSI discrepancies were greater than 1.0 in both the WAIS-III and WAIS-IV subgroups, although the specific discrepancy reaching statistical significance differed between versions (Supplementary Table 4).

**Table 3:** Odds ratios for low %EWL (%EWL ≥ 50%, 0; %EWL < 50%, 1)

	OR	95% CI	*P* value
Sex (male, 0; female, 1)	0.41	0.16–1.04	0.0598
Body weight (kg)	0.99	0.96–1.02	0.4648
BMI (kg/m^2^)	1.07	0.98–1.18	0.1141
VCI score (< 90, 0; ≥ 90, 1)	1.50	0.70–3.34	0.2999
Patients with WAIS index score discrepancy			
PRI–PSI (no, 0; yes, 1)	2.09	1.05–4.22	0.0346
WMI–PSI (no, 0; yes, 1)	1.97	1.01–3.91	0.0482

Akaike’s Information Criterion: 222.1; model: r^2^ = 0.1119, P = 0.0002

*OR* odds ratio, *EWL* excess weight loss, *BMI* body mass index, *WAIS* Wechsler Adult Intelligence Scale, *VCI* verbal comprehension index, *PRI *perceptual reasoning index, *PSI* processing speed index, *WMI *working memory index

### Comparison of patient characteristics between the PRI > PSI and PRI < PSI groups among patients with WAIS index score discrepancies between PRI and PSI

Two categories of patients had WAIS index score discrepancies between the PRI and PSI: PRI > PSI and PRI < PSI. Therefore, we compared patient characteristics between patients with PRI > PSI and PRI < PSI among patients with WAIS index score discrepancies between PRI and PSI (n = 92). In total, 64 (69.6%) and 28 (30.4%) patients were included in the PRI > PSI and PRI < PSI groups, respectively. Significant differences were observed in sex distribution, BW, and %EWL. The proportion of males, BW, and PRI score were significantly higher in the PRI > PSI group than in the PRI < PSI group, whereas the PSI score was significantly lower in the PRI > PSI group. The proportion of patients with %EWL < 50% post-LSG did not differ between the two groups; however, %EWL was significantly lower in the PRI > PSI group than in the PRI < PSI group (Supplementary Table 5). Other parameters had no significant between-group differences.

### Comparison of patient characteristics between the WMI > PSI and WMI < PSI groups among patients with WAIS index score discrepancy between WMI and PSI

Two categories of patients also had WAIS index score discrepancies between WMI and PSI: WMI > PSI and WMI < PSI. Therefore, we similarly compared patient characteristics between patients with WMI > PSI and those with WMI < PSI among those with WAIS index score discrepancies between WMI and PSI (n = 83). In total, 50 (60.2%) and 33 (39.8%) patients were included in the WMI > PSI and WMI < PSI groups, respectively. Age and WMI score were significantly higher in the WMI > PSI group than in the WMI < PSI group, whereas PSI score was significantly lower in the WMI > PSI group. However, neither %EWL nor the proportion of patients with %EWL < 50% post-LSG significantly differed between groups (Supplementary Table 6). Other parameters had no significant between-group differences.

### Comparison of patient characteristics, %EWL, and the proportion of patients with %EWL < 50% or ≥ 50% across each WAIS index score discrepancy patterns involving PRI, WMI, and PSI.

Some patients exhibited both WAIS index score discrepancies (PRI–PSI and WMI–PSI); therefore, we compared patients with two discrepancies (PRI–PSI and WMI–PSI) with those with one discrepancy (PRI–PSI or WMI–PSI). Although %EWL did not differ between groups, the proportion of patients with %EWL < 50% post-LSG was significantly higher in patients with two discrepancies (PRI–PSI and WMI–PSI; n = 43) than in those with one discrepancy (PRI–PSI or WMI–PSI; n = 89) (Table 4).

**Table 4:** Comparison between participants with one WAIS index score discrepancy (PRI–PSI or WMI–PSI) and those with two discrepancies (PRI–PSI and WMI–PSI)

	PRI–PSI or WMI–PSI	PRI–PSI and WMI–PSI	*P* value
No. of participants	89 (67.4%)	43 (32.6%)	
Sex (male/female)	44 (49.4%)/45 (50.6%)	24 (55.8%)/19 (44.2%)	0.5782^a^
Age (years)	45.0 (36.5–50.0)	44.0 (39.0–50.0)	0.6814^b^
Patients with mental disorders	50 (56.2%)	26 (60.5%)	0.7089^a^
BW (kg)	118.0 (101.4–147.5)	123.9 (108.3–136.5)	0.8555^b^
BMI (kg/m^2^)	44.1 (38.7–51.7)	44.2 (39.4–53.2)	0.8976^b^
%EWL (%)	63.1 (50.1–83.0)	57.8 (39.7–79.8)	0.2361^b^
%EWL < 50%	22 (24.7%)	20 (46.5%)	0.0165^a^
Patterns of WAIS index score discrepancy			
PRI > PSI/PRI < PSI	54 (60.7%)/35 (39.3%)	29 (67.4%)/14 (32.6%)	0.5647^a^
WMI > PSI/WMI < PSI	52 (58.4%)/37 (41.6%)	29 (67.4%)/14 (32.6%)	0.3463^a^

WAIS score discrepancies between patients with one WAIS index score discrepancy (PRI–PSI or WMI–PSI) and those with two discrepancies (PRI–PSI and WMI–PSI) (n = 132) were analyzed

Data are presented as medians, interquartile ranges (IQR), or percentages

^a^Fisher’s exact test; ^b^Wilcoxon rank-sum test

*WAIS* Wechsler Adult Intelligence Scale, *PRI* perceptual reasoning index, *WMI* working memory index, *PSI* processing speed index, *BW* body weight, *BMI* body mass index, *EWL* excess weight loss

Notably, the median %EWL was > 55% and the proportion of patients with %EWL ≥ 50% was > 50% when discrepancies existed between PRI/WMI and PSI (Table 5).

**Table 5:** %EWL and the proportion of patients with %EWL ≥ 50% among patients with WAIS index score discrepancies (PRI–PSI and/or WMI–PSI)

	%EWL	%EWL ≥ 50%
WAIS index score discrepancy		
PRI–PSI (n = 92)	59.9 (46.1–79.5)	59 (64.1%)
WMI–PSI (n = 83)	64.0 (43.8–81.7)	54 (65.1%)
PRI–PSI or WMI–PSI (n = 132)	62.8 (46.8–81.2)	90 (68.2%)
PRI–PSI and WMI–PSI (n = 43)	57.8 (39.7–79.8)	23 (53.5%)

Data are presented as medians, interquartile ranges (IQR), or percentages

*EWL* excess weight loss, *WAIS* Wechsler Adult Intelligence Scale, *PRI* perceptual reasoning index, *WMI *working memory index, *PSI* processing speed index

## Discussion

In this study, patients who underwent LSG demonstrated significant improvements in BW and BMI after 12 months. No relationship was observed between preoperative BMI and IQ, including WAIS index scores and WAIS index score discrepancies. However, the prevalence of the two types of WAIS index score discrepancies (PRI–PSI and WMI–PSI) was significantly higher in patients with %EWL < 50%. Multivariate logistic regression analyses identified independent associations between both WAIS discrepancies (PRI–PSI and WMI–PSI) and lower %EWL post-LSG. Furthermore, the proportion of patients with %EWL < 50% post-LSG was significantly higher among patients with two WAIS index score discrepancies (PRI–PSI and WMI–PSI) than among those with one discrepancy (PRI–PSI or WMI–PSI).

In this study, two types of WAIS score discrepancies (PRI–PSI and WMI–PSI) were associated with insufficient weight reduction post-LSG. PSI was an important WAIS index in this study because patients with lower PSI scores had lower EWL post-LSG among those with WAIS index score discrepancies between PRI and PSI. A lower processing speed is associated with obesity [28] and with more severe general mental health symptoms and neurodevelopmental disorders [29, 30]. Further, patients with obesity and developmental disorders exhibited insufficient weight loss post-LSG [31].

Obesity influences the brain structure, which in turn influences IQ scores. Specifically, obesity is associated with reduced white and gray matter volumes [32, 33]. A higher BMI is associated with decreased fractional anisotropy in white matter fibers connecting brain regions that support working memory, potentially contributing to poor working memory performance in individuals with obesity [34]. Furthermore, WMI has been positively correlated with white matter, gray matter, and cerebellar volumes [35]. Similarly, PSI is positively related to white matter volume, and PRI positively correlates with cerebellar volume [35]. However, VCI is not associated with any of these brain volumes [35]. IQ discrepancy also influences brain structure, and discrepancies between verbal and performance IQ have been associated with cortical thickness in the brain [36]. In addition, patterns of IQ or WAIS index score discrepancies have been associated with specific brain structures or lesion locations [36, 37]. In this study, WAIS index score discrepancies were related to insufficient weight reduction post-LSG, whereas VCI was not. Therefore, our findings may reflect alterations in neural systems related to perceptual reasoning, working memory, and processing speed, such as frontoparietal networks and white matter integrity, which have been previously linked to obesity and WAIS subscale performance.

One possible mechanism underlying the association between WAIS index score discrepancies and insufficient weight reduction after LSG involves postoperative self-management capacity. Processing speed reflects the ability to efficiently translate cognitive understanding into action [38], which is essential for sustained behavioral change after bariatric surgery. Patients with relatively lower PSI compared with PRI or WMI may understand postoperative recommendations but have difficulty implementing them consistently in daily life. From this perspective, WAIS index score discrepancies may reflect cognitive imbalance rather than global intellectual impairment, leading to a gap between knowing "what should be done" and actually "doing it". Neurobiologically, such discrepancies may be related to obesity-associated alterations in brain regions supporting processing speed and executive function [32, 35], as PSI is linked to white matter integrity, whereas PRI and WMI involve partly distinct neural networks [35].

It should be emphasized that the present study does not allow causal inference regarding the relationship between obesity and cognitive characteristics. The association observed between WAIS index score discrepancies and insufficient weight loss may reflect a bidirectional relationship. Obesity may contribute to alterations in brain structure and cognitive performance, while pre-existing cognitive characteristics—particularly reduced processing speed—may influence health-related behaviors, adherence to postoperative recommendations, and weight loss outcomes. Importantly, these findings should not be interpreted as indicating irreversible cognitive impairment in patients with obesity. A previous study has demonstrated that processing speed can improve after bariatric surgery [39]. Because postoperative WAIS assessments were not performed in this study, we were unable to evaluate changes in cognitive function after surgery. Therefore, whether WAIS index score discrepancies improve following weight loss remains an important topic for future prospective studies.

Although two types of WAIS score discrepancies (PRI–PSI and WMI–PSI) remained significantly associated with insufficient weight loss after LSG, r^2^ values of the multivariate logistic regression models were modest. Therefore, the predictive strength of the models is limited, and the findings should be interpreted as exploratory rather than definitive.

Although both types of WAIS index score discrepancies (PRI–PSI and WMI–PSI) were associated with insufficient weight reduction post-LSG, these index scores may improve. In individuals with obesity, aerobic fitness is associated with improved working memory and processing speed [40]. Further, Roux-en-Y gastric bypass and gastric banding reportedly improve processing speed 6 months postoperatively [39]. These previous reports indicate WAIS index score discrepancies may be amenable to intervention.

This study has several limitations. First, although bariatric surgery improves processing speed and neurocognitive performance [39, 41], no postoperative IQ tests were conducted. Therefore, the postoperative IQ scores remained unknown. However, both WAIS-III and IV have practical effects, whereby prior exposure to WAIS-III or IV significantly increases scores [42, 43]. Therefore, the reliability of postoperative IQ test results must be considered. Second, although all patients received monthly structured nutritional counselling from registered dietitians, detailed quantitative assessments of individual dietary habits were not collected. Likewise, standardized quantitative physical examination parameters (e.g., physical activity level or muscle strength) were not recorded and therefore could not be included as confounders. Furthermore, details on education and adherence were unavailable, preventing adjustment for these factors. Therefore, residual confounding related to socioeconomic or behavioral factors could not be excluded. In addition, although the gastric sleeve was created using a uniform surgical technique in all cases (gastric transection 5 cm proximal to the pylorus using a 36-Fr calibration tube), postoperative gastric remnant volume was not directly measured. Therefore, residual variability in these unmeasured confounding factors cannot be completely excluded. Third, although our institution performs IQ testing in a large proportion of bariatric candidates, the sample size remained relatively small. This is primarily because the prevalence of severe obesity is lower in Japan than in Western countries, resulting in a limited annual volume of bariatric procedures. Consequently, even with a long inclusion period, the number of eligible patients did not reach that seen in high-volume international centers. In addition, the retrospective design limits our ability to control for potential confounders, introduces the risk of selection and information bias due to reliance on past records, and prevents causal inference. Moreover, we did not include a control group of individuals with obesity who underwent IQ testing but did not undergo surgery, which prevents us from determining whether similar weight trajectories might have occurred without LSG. Future prospective multicenter studies with larger and more diverse cohorts and appropriate non-surgical comparison groups are warranted to validate and extend our findings. Fourth, a new standardized definition of suboptimal clinical response (%total weight loss < 20%) was proposed in 2024 [44]. As our study was designed and conducted before this update, insufficient weight loss was defined a priori as %EWL < 50%, which was the conventional criterion in previous bariatric surgery research. Future prospective studies incorporating %Total Weight Loss-based definitions from the planning stage are warranted to confirm the generalizability of our findings. Fifth, both the WAIS-III and WAIS-IV were used because our institution transitioned from WAIS-III to WAIS-IV during the study period. Although these versions share similar index structures, combining them may introduce measurement variability and limit the generalizability of the findings to other populations. Sixth, the prevalence of psychiatric comorbidities in our cohort was high, which may not reflect the characteristics of other bariatric populations. Such comorbidities could influence cognitive function and postoperative behavioral adherence, thereby affecting weight loss outcomes. Seventh, postoperative cognitive assessments were not conducted, preventing us from evaluating whether WAIS index discrepancies change after substantial weight reduction. Consequently, causal inference between cognitive profiles and insufficient weight loss should be interpreted cautiously. Finally, although weight regain may occur beyond the second postoperative year, the present study focused on weight loss outcomes at 12 months, which reflects the primary surgical effect. Future prospective studies with longer follow-up periods are warranted to determine whether WAIS index score discrepancies are also associated with long-term weight maintenance or weight regain after LSG. Despite these limitations, we demonstrated the relationship between WAIS index score discrepancies and insufficient weight reduction following LSG in adults with obesity.

## Conclusion

The presence of a WAIS index discrepancy between PRI and PSI or between WMI and PSI is associated with insufficient weight reduction following LSG in adults with obesity. Furthermore, the rate of “good” weight loss results (%EWL ≥ 50%) post-LSG is lower among patients with both WAIS index score discrepancies (PRI–PSI and WMI–PSI). Therefore, preoperative IQ tests may be useful in predicting insufficient weight loss post-LSG. These findings indicate an association—not a causal relationship—between WAIS index discrepancies and postoperative weight loss. Whether cognitive characteristics contribute to reduced weight loss, or whether both reflect underlying factors not measured in this study, remains unknown. Therefore, WAIS testing may serve as a supportive clinical tool, but it should not be interpreted as evidence of causality or used as an exclusion criterion.

## Supplementary Information

Supplementary file 1

Supplementary file 2

## Data Availability

The data that support the findings of this study are not publicly available due to privacy reason but are available from the corresponding author upon reasonable request.

## Abbreviations

LSG: Laparoscopic sleeve gastrectomy
EWL: Excess weight loss
IQ: Intelligence quotient
BMI: Body mass index
BW: Body weight
WAIS: Wechsler Adult Intelligence Scale
FBG: Fasting blood glucose
HbA1c: Glycosylated hemoglobin
VCI: Verbal comprehension index
PRI: Perceptual reasoning index
WMI: Working memory index
PSI: Processing speed index
IQR: Interquartile ranges
ORs: Odds ratios
CIs: Confidence intervals
