# Supplementary material for: Two types of Wechsler Adult Intelligence Scale (WAIS) index discrepancies are associated with insufficient weight reduction after sleeve gastrectomy in adult patients with obesity: a retrospective study
Source: Eur J Med Res. 2026 Jan 6;31:203. doi: 10.1186/s40001-025-03758-y (PMC12869949; doi:10.1186/s40001-025-03758-y)
Supplement: Supplementary file 1 [file 40001_2025_3758_MOESM1_ESM.docx]

Supplementally Table 1. Comparisons of baseline characteristics between included and excluded patients in this study

|  | Included patients (n = 204) | | Excluded patients (n = 51) | *P* value |
| --- | --- | --- | --- | --- |
| Sex (male/female)  Age (years)  Type 2 diabetes mellitus  Preoperative BW (kg)  Preoperative BMI (kg/m^2^)  Patients with mental disorders | | 95 (46.6%)/109 (53.4%)  44.5 (37.0–50.0)  120 (58.8%)  117.9 (101.2–138.7)  43.6 (38.7–50.4)  123 (60.3%) | 22(43.1%)/29(56.9%)  44.0 (36.5–53.5)  28 (54.9%)  113.1 (98.7–133.8)  45.0 (37.6–51.4)  28 (54.9%) | 0.7538^a^  0.6255^b^  0.6366^a^  0.5822^b^  0.7094^b^  0.5256^a^ |

Data are presented as medians, interquartile ranges (IQR), or percentages.

^a^Fisher’s exact test; ^b^Wilcoxon rank-sum test.

LSG, laparoscopic sleeve gastrectomy; EWL, excess weight loss; IQ, intelligence quotient; BW, body weight; BMI, body mass index.

Supplementary Table 2. Comparison of characteristics and IQ examination between patients without and with mental disorders

|  | Patients without mental disorders | Patients with  mental disorders | *P* value |
| --- | --- | --- | --- |
| No. of participants  Sex (male/female)  Age (years)  Patients with Mental or Developmental retardation  Type 2 diabetes mellitus  BW (kg)  BMI (kg/m^2^)  %EWL (%)  %EWL < 50%  Full-Scale IQ  VCI score  PRI score  WMI score  PRI score  Patients with WAIS index score discrepancy  VCI–PRI  VCI–WMI  VCI–PSI  PRI–WMI  PRI–PSI  WMI–PSI | 81 (39.7%)  50 (61.7%)/31 (38.3%)  45.0 (37.5–50.5)  0 (0%)  48 (59.3%)  122.4 (100.8–141.0)  43.0 (37.3–51.0)  66.5 (51.7–86.2)  18 (22.2%)  96.0 (85.5–105.5)  97.0 (87.0–108.0)  97.0 (85.0–108.5)  94.0 (83.5–105.0)  89.0 (81.0–100.0)  31 (38.3%)  43 (53.1%)  41 (50.6%)  43 (53.1%)  36 (44.4%)  37 (45.7%) | 123 (60.3%)  45 (36.6%)/78 (63.4%)  44.0 (36.0–50.0)  22 (17.9%)  72 (58.5%)  115.0 (101.2–136.8)  43.9 (38.9–50.2)  63.1 (46.9–83.3)  35 (28.5%)  89.0 (79.0–102.0)  95.0 (81.0–104.0)  93.0 (79.0–105.0)  91.0 (81.0–100.0)  87.0 (79.0–97.0)  51 (41.5%)  51 (41.5%)  57 (46.3%)  45 (36.6%)  56 (45.5%)  46 (37.4%) | 0.0006^a^  0.5204^b^  <0.0001^a^  1.0000^a^  0.3789^b^  0.5880^b^  0.2173^b^  0.3338^a^  0.0356^b^  0.4187^b^  0.0497^b^  0.0865^b^  0.1709^b^  0.6645^a^  0.1157^a^  0.3914^a^  0.1113^a^  0.8868^a^  0.2478^a^ |

Data are presented as medians, interquartile ranges (IQR), or percentages.

^a^Fisher’s exact test; ^b^Wilcoxon rank-sum test.

BW, body weight; BMI, body mass index; EWL, excess weight loss; IQ, intelligence quotient; WAIS, Wechsler Adult Intelligence Scale; VCI, verbal comprehension index; PRI, perceptual reasoning index; WMI, working memory index; PSI, processing speed index.

Supplementary Table 3. Comparison of characteristics between patients with postoperative BMI <43.6 kg/m^2^ and ≥43.6 kg/m^2^

|  | BMI <43.6 kg/m^2^ | BMI ≥43.6 kg/m^2^ | *P* value |
| --- | --- | --- | --- |
| No. of participants  Sex (male/female)  Age (years)  Type 2 diabetes mellitus  Patients with mental disorders  BW (kg)  BMI (kg/m^2^)  %EWL (%)  %EWL < 50%  Full-Scale IQ  VCI score  PRI score  WMI score  PSI score  Patients with WAIS index score discrepancy  VCI–PRI  VCI–WMI  VCI–PSI  PRI–WMI  PRI–PSI  WMI–PSI | 102 (50.0%)  35 (34.3%)/67 (65.7%)  44.0 (36.0–51.0)  61 (59.8%)  59 (57.8%)  101.4 (93.1–113.4)  38.7 (36.2–41.0)  74.8 (58.7–95.8)  17 (16.7%)  90.0 (81.0–103.0)  95.0 (79.0–104.5)  95.0 (83.0–106.0)  91.0 (82.0–100.0)  89.5 (79.0–100.0)  41 (40.2%)  48 (47.1%)  52 (51.0%)  36 (35.3%)  42 (41.2%)  40 (39.2%) | 102 (50.0%)  60 (58.8%)/42 (41.2%)  45.0 (37.8–49.0)  59 (57.8%)  64 (62.7%)  136.1 (124.3–161.2)  50.4 (45.8–55.5)  57.1 (42.4–73.7)  36 (35.3%)  92.0 (82.8–104.3)  97.0 (88.0–107.0)  93.0 (82.0–107.0)  94.0 (81.0–103.0)  87.0 (80.5–94.5)  41 (40.2%)  46 (45.1%)  48 (47.1%)  48 (47.1%)  50 (49.0%)  43 (42.2%) | 0.0007^a^  0.7685^b^  0.8869^a^  0.5672^a^  <0.0001^b^  <0.0001^b^  <0.0001^b^  0.0038^a^  0.3660^b^  0.2517^b^  0.8096^b^  0.4595^b^  0.1575^b^  1.0000^a^  0.8883^a^  0.6745^a^  0.1174^a^  0.3247^a^  0.7757^a^ |

Data are presented as medians, interquartile ranges (IQR), or percentages.

^a^Fisher’s exact test; ^b^Wilcoxon rank-sum test.

BW, body weight; BMI, body mass index; EWL, excess weight loss; IQ, intelligence quotient; WAIS, Wechsler Adult Intelligence Scale; VCI, verbal comprehension index; PRI, perceptual reasoning index; WMI, working memory index; PSI, processing speed index.

Supplementary Table 4

A. Odds ratios for low %EWL (%EWL ≥50%, 0; %EWL <50%, 1) in patients who underwent WAIS-III (n = 86)

|  | OR | 95% CI | *P* value |
| --- | --- | --- | --- |
| Sex (male, 0; female, 1)  Body weight (kg)  BMI (kg/m^2^)  VCI score (<90, 0; ≥90, 1)  Patients with WAIS index score discrepancy  PRI–PSI (no, 0; yes, 1)  WMI–PSI (no, 0; yes, 1) | 0.71  1.01  1.05  0.70  3.72  1.15 | 0.17–3.03  0.97–1.06  0.91–1.22  0.21–2.38  1.13–14.09  0.38–3.47 | 0.6443  0.6175  0.4812  0.5594  0.0298  0.8002 |

Note: Akaike’s Information Criterion: 97.6; model: r^2^ = 0.1597, P = 0.0159.

OR: odds ratio; EWL: excess weight loss; BMI: body mass index; WAIS, Wechsler Adult Intelligence Scale; VCI: verbal comprehension index; PRI: perceptual reasoning index; PSI: processing speed index; WMI: working memory index.

B. Odds ratios for low %EWL (%EWL ≥50%, 0; %EWL <50%, 1) in patients who underwent WAIS-IV (n = 118)

|  | OR | 95% CI | *P* value |
| --- | --- | --- | --- |
| Sex (male, 0; female, 1)  Body weight (kg)  BMI (kg/m^2^)  VCI score (<90, 0; ≥90, 1)  Patients with WAIS index score discrepancy  PRI–PSI (no, 0; yes, 1)  WMI–PSI (no, 0; yes, 1) | 0.21  0.95  1.14  3.16  1.76  3.15 | 0.05–0.82  0.90–0.99  1.01–1.30  1.07–10.92  0.69–4.49  1.25–8.27 | 0.0246  0.0406  0.0390  0.0372  0.2355  0.0152 |

Note: Akaike’s Information Criterion: 128.9; model: r^2^ = 0.1618, P = 0.0012.

OR: odds ratio; EWL: excess weight loss; BMI: body mass index; WAIS, Wechsler Adult Intelligence Scale; VCI: verbal comprehension index; PRI: perceptual reasoning index; PSI: processing speed index; WMI: working memory index.

Supplementary Table 5. Comparison between the PRI > PSI and PRI < PSI groups among patients with WAIS index score discrepancies between PRI and PSI

|  | PRI > PSI | PRI < PSI | *P* value |
| --- | --- | --- | --- |
| No. of participants  Sex (male/female)  Age (years)  Type 2 diabetes mellitus  Patients with mental disorders  BW (kg)  BMI (kg/m^2^)  %EWL (%)  %EWL < 50%  Full-Scale IQ  VCI score  PRI score  WMI score  PSI score  Patients with WAIS index score discrepancy  VCI–PRI  VCI–WMI  VCI–PSI  PRI–WMI  WMI–PSI | 64 (69.6%)  40 (62.5%)/24 (37.5%)  45.0 (40.0–50.8)  37 (57.8%)  39 (60.9%)  128.7 (109.3–153.1)  44.9 (39.7–53.5)  58.0 (43.7–70.6)  24 (37.5%)  98.5 (87.5–109.5)  97.5 (92.0–111.8)  107.0 (97.5–114.0)  97.0 (85.0–108.5)  85.0 (75.0–92.0)  30 (46.9%)  34 (53.1%)  40 (62.5%)  32 (50.0%)  29 (45.3%) | 28 (30.4%)  8 (28.6%)/20 (71.4%)  42.5 (345–50.0)  19 (67.9%)  17 (60.7%)  107.6 (95.1–117.4)  41.5 (38.3–48.2)  72.9 (47.9–94.3)  9 (32.1%)  90.0 (72.5–105.8)  95.0 (73.0–109.3)  82.5 (69.5–97.0)  91.5 (81.3–100.0)  102.0 (90.5–121.0)  12 (42.9%)  12 (42.9%)  18 (64.3%)  13 (46.4%)  14 (50.0%) | 0.0033^a^  0.2073^b^  0.4869^a^  1.0000^a^  0.0006^b^  0.0720^b^  0.0443^b^  0.8136^a^  0.0504^b^  0.0952^b^  <0.0001^b^  0.0994^b^  <0.0001^b^  0.8214^a^  0.4971^a^  1.0000^a^  0.8227^a^  0.8208^a^ |

Participants with WAIS score discrepancies between the PRI and PSI (n = 92) were analyzed. Data are presented as medians, interquartile ranges (IQR), or percentages.

^a^Fisher’s exact test; ^b^Wilcoxon rank-sum test.

WAIS, Wechsler Adult Intelligence Scale; PRI, perceptual reasoning index; PSI, processing speed index; BW, body weight; BMI, body mass index; EWL, excess weight loss; IQ, intelligence quotient; VCI, verbal comprehension index; WMI, working memory index.

Supplementary Table 6. Comparison between the WMI > PSI and WMI < PSI groups among participants with WAIS index score discrepancies between WMI and PSI

|  | WMI > PSI | WMI < PSI | *P* value |
| --- | --- | --- | --- |
| No. of participants  Sex (male/female)  Age (years)  Type 2 diabetes mellitus  Patients with mental disorders  BW (kg)  BMI (kg/m^2^)  %EWL (%)  %EWL < 50%  Full-Scale IQ  VCI score  PRI score  WMI score  PSI score  Patients with WAIS index score discrepancy  VCI–PRI  VCI–WMI  VCI–PSI  PRI–WMI  PRI–PSI | 50 (60.2%)  31 (62.0%)/19 (38.0%)  47.0 (39.0–51.3)  33 (66.0%)  28 (56.0%)  128.7 (107.6–153.0)  45.2 (38.8–53.2)  64.2 (43.6–77.6)  17 (34.0%)  93.5 (83.0–108.0)  98.5 (87.5–110.0)  93.0 (80.8–111.5)  101.0 (94.0–115.5)  82.0 (73.0–89.3)  27 (54.0%)  22 (44.0%)  35 (70.0%)  28 (56.0%)  29 (58.0%) | 33 (39.8%)  13 (39.4%)/20 (60.6%)  39.0 (34.0–48.5)  27 (81.8%)  18 (54.5%)  113.2 (103.6–135.2)  41.9 (38.7–53.1)  63.1 (43.4–90.9)  12 (36.4%)  96.0 (78.5–105.5)  93.0 (77.0–108.0)  97.0 (81.0–104.5)  83.0 (71.5–95.5)  100.0 (89.5–114.0)  11 (33.3%)  18 (54.5%)  16 (48.5%)  16 (48.5%)  14 (42.4%) | 0.0715^a^  0.0169^b^  0.1383^a^  1.0000^a^  0.1224^b^  0.5640^b^  0.4940^b^  1.0000^a^  0.4480^b^  0.0739^b^  0.7339^b^  <0.0001^b^  <0.0001^b^  0.0754^a^  0.3772^a^  0.0659^a^  0.6535^a^  0.1848^a^ |

Participants with WAIS score discrepancies between the WMI and PSI (n = 83) were analyzed.

Data are presented as medians, interquartile ranges (IQR), or percentages.

^a^Fisher’s exact test; ^b^Wilcoxon rank-sum test.

WAIS, Wechsler Adult Intelligence Scale; WMI, working memory index; PSI, processing speed index; BW, body weight; BMI, body mass index; EWL, excess weight loss; IQ, intelligence quotient; VCI, verbal comprehension index; PRI, perceptual reasoning index.
