# Supplementary material for: Two types of Wechsler Adult Intelligence Scale (WAIS) index discrepancies are associated with insufficient weight reduction after sleeve gastrectomy in adult patients with obesity: a retrospective study
Source: Eur J Med Res. 2026 Jan 6;31:203. doi: 10.1186/s40001-025-03758-y (PMC12869949; doi:10.1186/s40001-025-03758-y)
Supplement: Supplementary file 2 [file 40001_2025_3758_MOESM2_ESM.docx]

**Supplementary file 1**

**Verbal comprehension index (VCI), perceptual reasoning index (PRI), working memory index (WMI), and processing speed index (PSI)**

VCI refers to the ability to verbally express thoughts and understand, analyze, and interpret written information. PRI refers to nonverbal reasoning, spatial processing, and visual perception. WMI refers to the ability to simultaneously hold several pieces of information in the mind and manipulate and use this information. PSI refers to visual perception, scanning, and hand-eye coordination [23].
